# Dual-energy CT kidney stone characterization—can diagnostic accuracy be achieved at low radiation dose?

**DOI:** 10.1007/s00330-023-09569-1

**Published:** 2023-03-29

**Authors:** André Euler, Sara Wullschleger, Thomas Sartoretti, Daniel Müller, Etienne Xavier Keller, Dejan Lavrek, Olivio Donati

**Affiliations:** 1 Diagnostic and Interventional Radiology, University Hospital Zurich, University of Zurich, Rämistrasse 100, 8091 Zurich, Switzerland; 2 Institute of Clinical Chemistry, University Hospital Zurich, University of Zurich, Rämistrasse 100, 8091 Zurich, Switzerland; 3 Department of Urology, University Hospital Zurich, University of Zurich, Zurich, Switzerland

**Keywords:** Computed tomography, X-ray, Urolithiasis, Kidney calculi, X-ray diffraction

## Abstract

**Objectives:**

To assess the accuracy of low-dose dual-energy computed tomography (DECT) to differentiate uric acid from non-uric acid kidney stones in two generations of dual-source DECT with stone composition analysis as the reference standard.

**Methods:**

Patients who received a low-dose unenhanced DECT for the detection or follow-up of urolithiasis and stone extraction with stone composition analysis between January 2020 and January 2022 were retrospectively included. Collected stones were characterized using X-ray diffraction. Size, volume, CT attenuation, and stone characterization were assessed using DECT post-processing software. Characterization as uric acid or non-uric acid stones was compared to stone composition analysis as the reference standard. Sensitivity, specificity, and accuracy of stone classification were computed. Dose length product (DLP) and effective dose served as radiation dose estimates.

**Results:**

A total of 227 stones in 203 patients were analyzed. Stone composition analysis identified 15 uric acid and 212 non-uric acid stones. Mean size and volume were 4.7 mm × 2.8 mm and 114 mm^3^, respectively. CT attenuation of uric acid stones was significantly lower as compared to non-uric acid stones (*p* < 0.001). Two hundred twenty-five of 227 kidney stones were correctly classified by DECT. Pooled sensitivity, specificity, and accuracy were 1.0 (95%CI: 0.97, 1.00), 0.93 (95%CI: 0.68, 1.00), and 0.99 (95%CI: 0.97, 1.00), respectively. Eighty-two of 84 stones with a diameter of  ≤ 3 mm were correctly classified. Mean DLP was 162 ± 57 mGy*cm and effective dose was 2.43 ± 0.86 mSv.

**Conclusions:**

Low-dose dual-source DECT demonstrated high accuracy to discriminate uric acid from non-uric acid stones even at small stone sizes.

**Key Points:**

• *Two hundred twenty-five of 227 stones were correctly classified as uric acid vs. non-uric acid stones by low-dose dual-energy CT with stone composition analysis as the reference standard*.

• *Pooled sensitivity, specificity, and accuracy for stone characterization were 1.0, 0.93, and 0.99, respectively*.

• *Low-dose dual-energy CT for stone characterization was feasible in the majority of small stones * < *3 mm*.

## Introduction

Computed tomography (CT) has been routinely implemented as the reference imaging test to rule out urolithiasis, particularly in the emergency setting [1]. The use of CT in patients with suspected urolithiasis has substantially increased in the last two decades [2]. In addition, urolithiasis tends to relapse [3, 4], leading to repetitive imaging and consecutively increasing patients’ cumulative exposure to ionizing radiation.

Therefore, low-dose single-energy CT has been implemented and has been shown to rule out urolithiasis (U-CT) with high accuracy [5]. While the definition of a “low-dose” CT is often arbitrary, a dose length product (DLP) of  < 200 mGy*cm has generally been accepted as the threshold for low-dose U-CT [6]. Unfortunately, there are few studies focusing on the use of low-dose U-CT in routine clinical practice [6, 7].

Dual-energy CT (DECT) offers an alternative to single-energy CT due to its ability to differentiate uric acid from non-uric acid stones. This discrimination can guide therapeutic decision-making as uric acid stones can be treated pharmacologically by alkalization of the urine and stone recurrence can be influenced by metaphylaxis and secondary prevention [8, 9]. Two meta-analyses have found high accuracy of DECT for stone characterization [10, 11] with a mean pooled sensitivity of 82% and 95.5% and a specificity of 97% and 98.5%, respectively. Furthermore, recent phantom studies demonstrated high accuracy among different DECT approaches [12, 13]. Decreased accuracy for stone characterization with DECT has only been reported for small stones  < 3 mm [12, 14] and for stones  < 5 mm at reduced radiation dose [15].

Nevertheless, several former DECT studies were limited by their design as phantom studies [12, 13, 16, 17], by the use of DECT as a stone-targeted approach [18, 19], i.e. a limited scan range, or by the lack or limited availability of an ex vivo stone composition analysis as the reference standard in all stones [15, 20].

Based on the high accuracy of DECT and the increased awareness to reduce radiation dose exposure, it is desirable to establish DECT combined with low-dose technique as the reference in U-CT. Therefore, the purpose of our in vivo study was to assess the accuracy of low-dose DECT to differentiate between uric acid and non-uric acid stones for two generations of dual-source DECT with stone composition analysis as the reference standard.

## Materials and methods

### Patient selection

This single-center retrospective study was conducted at an academic medical center and received institutional review board and local ethics committee approval. Patients 18 years or older who received an unenhanced single-phase DECT on one of two dual-source DECTs for the detection or follow-up of urolithiasis between January 2020 and January 2022 were retrospectively identified (*n* = 818). Patients were excluded if urolithiasis was absent (*n* = 312), if more than 5 kidney stones were present (*n* = 5) or if dual-energy datasets for post-processing were not available (*n* = 3). Of the remaining patients (*n* = 498), only patients for which an interventional stone extraction (ureteroscopy or percutaneous nephrolithotomy) and stone composition analysis were performed within 6 months from imaging were included (*n* = 203). The anteroposterior diameter (AD) and lateral diameter (LD) of each patient at the level of the mid-abdomen was measured and the effective diameter (ED) was calculated as ED = √[AD·LD].

### CT scan protocol

Imaging was performed on either of the two CT scanners installed at our institution: (A) a second-generation dual-source DECT (SOMATOM Flash, Siemens Healthcare GmbH) or (B) a third-generation dual-source DECT (SOMATOM Force, Siemens). Vendor-recommended protocols were used for which the quality reference tube current time product was adapted to achieve low-dose imaging. The acquisition parameters are summarized in Table 1. Image datasets were reconstructed as “mixed” series with a slice thickness of 2 mm and an increment of 1.6 mm and as low-energy and high-energy series with a slice thickness of 1.5 mm and an increment of 1 mm for dual-energy post-processing. A quantitative Qr40 reconstruction kernel was used for the post-processing reconstructions. An advanced modeled iterative reconstruction algorithm (ADMIRE, Siemens) at a strength level of three was applied. Volume CT dose index (CTDI_vol_), dose length product (DLP), and effective dose served as radiation dose estimates.

**Table 1:** CT scan parameters

CT parameter	2nd-generation DECT (A)	3rd-generation DECT (B)
Detector collimation (mm)	32 × 0.6	128 × 0.6
Tube voltage combination (kV)	100/Sn140	100/Sn150
Quality reference tube current-time product (ref. mAs)	80 (tube A) 62 (tube B)	80 (tube A) 40 (tube B)
Gantry rotation time (s)	0.5	0.5
Pitch	0.6	0.7
DLP (mGy*cm)	158 ± 47 [132, 177]	183 ± 93 [134, 186]
CTDI_vol_ (mGy)	4.18 ± 0.88 [3.54, 4.68]	4.21 ± 1.85 [3.28, 4.51]
Effective dose (mSv)	2.37 ± 0.7 [1.98, 2.02]	2.74 ± 1.4 [2.02, 2.8]

Automatic tube current modulation (ATCM) was used. Interquartile range is given in squared brackets. *Sn*, tin filtration; *DLP*, dose length product; *CTDIvol*, volume CT dose index

### Stone composition analysis

The clinical information system was reviewed for the extraction procedure report and stone analysis report. The reported side of the extraction, the number, and the size of the extracted stones were recorded. When multiple stones were located on the same side and only one stone was sent for analysis, the stone composition of this stone was attributed to all ipsilateral stones. Extracted stones were characterized using X-ray diffraction. Stones were crushed and analyzed using an X-ray diffractometer (Phaser D2, Bruker) by Ni-filtered CuK radiation. Identification of the constituents and semi-quantification by relative intensity was done using the PDF-4 + database (International Center for Diffraction Data). Categorization in compound stones was performed according to the main stone composition.

### Dual-energy CT stone analysis

Stone characterization was performed in each patient by a medical student (S.W.) using the default settings of a vendor-specific DECT post-processing workflow (syngo.CT Dual Energy, Kidney Stones, Syngo.via VB60A, Siemens Healthineers; resolution 3, minimum 200, maximum 3071). Readout was performed after a training session of 10 cases. The dual-energy workflow automatically discriminated uric acid from non-uric acid stones based on their dual-energy ratio. Uric acid stones were color-coded in red, while non-uric acid stones were coded in blue. For each stone, the size (as maximal diameter and orthogonal width), the volume, and the dual-energy ratio were recorded. If multiple stones of different compositions were present in the same patient, matching the imaging data to the stone composition analysis was ensured by comparing stone characteristics (side, volume) between CT and urological extraction report by a board-certified radiologist (A.E.) with 10 years of experience in abdominal imaging. In small stones, which were not automatically characterized by the software at the default setting, the resolution parameter of the algorithm was changed to a more sensitive value of 1.

### Statistical analysis

All statistical analyses were performed in the R programming language (version 4.0.2). Sensitivity, specificity, and accuracy of stone classification were calculated using ex vivo clinical stone analysis as the reference standard. To check for differences in the distribution or mean values between the two groups, Mann-Whitney *U* tests or Student’s *t*-tests were computed. Data are presented as mean ± standard deviation (SD) and/or median; [interquartile range (IQR)]. *p* values  < 0.05 were considered significant.

## Results

### Patient characteristics

A total of 203 patients (45 females, 158 men; 49 ± 15.1 years, interquartile range (IQR) [38.1, 56.8 years]) were included in this study. One hundred seventy-one and 32 patients were imaged on a CT scanner (A) and (B), respectively. The effective diameter of the patients was on average 29.2 ± 6.2 cm, IQR [25.8, 31.2 cm]. Mean DLP was 162 ± 57 mGy*cm, IQR [132, 179 mGy*cm]; CTDI_vol_ was 4.19 ± 1.08 mGy, IQR [3.53, 4.65 mGy]; and effective dose was 2.43 ± 0.86 mSv [1.98, 2.68 mSv]. Radiation dose parameters for each scanner are listed in Table 1.

### Stone characteristics

**Fig. 1:**
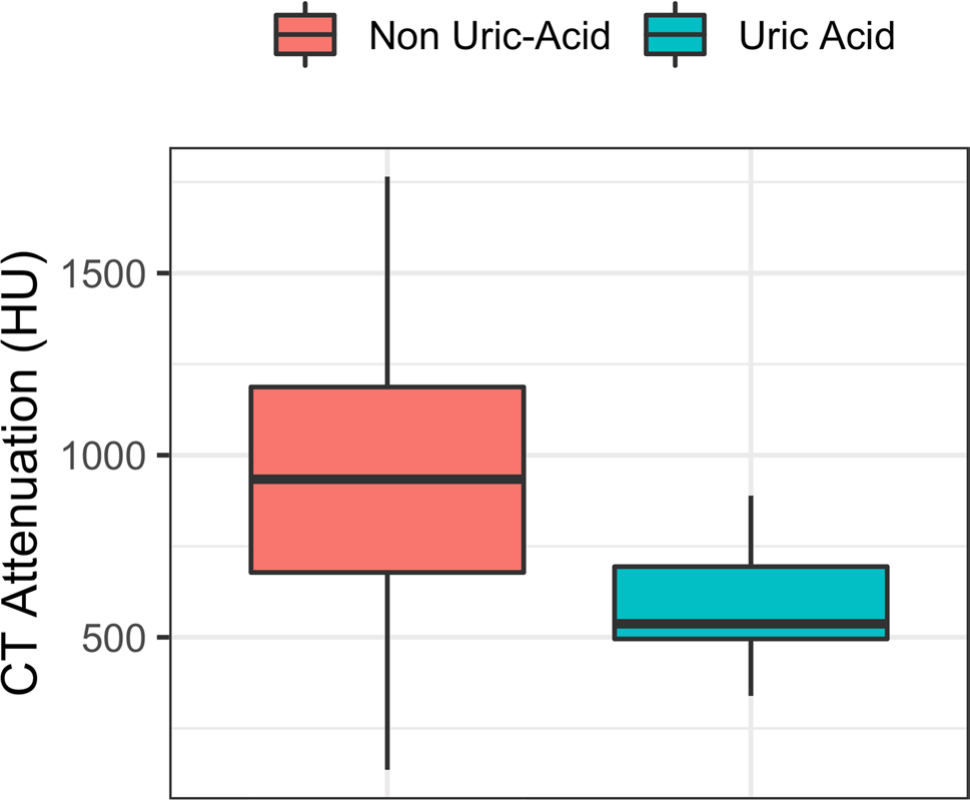
CT attenuation of kidney stones. Comparison of CT attenuation between non-uric acid and uric-acid stones. Note the increased CT attenuation of non-uric acid stones as compared to uric acid stones

A total of 227 kidney stones were analyzed. Of these stones, 191 and 36 were imaged on CT scanner (A) and (B), respectively. Stone composition analysis classified 15 stones as uric acid and 212 stones as non-uric acid. Of the non-uric acid stones, there were 175 calcium oxalate, 26 calcium oxalate – apatite, 4 cystine, 3 apatite, 2 calcium oxalate – calcium hydrogen phosphate, 1 triple phosphate, and 1 apatite – triple phosphate stone. CT attenuation of uric acid stones was significantly lower as compared to non-uric acid stones (*p* < 0.001) (Fig. 1). Additional descriptive stone characteristics are summarized in Table 2.

**Table 2:** Stone characteristics

	All stones	Uric acid	Non-uric acid	*p* value
Length (mm)	4.7 ± 3.3 [2.7, 5.4]	6.1 ± 4.5 [4.6, 6.1]	4.6 ± 3.2 [2.6, 5.3]	0.21
Width (mm)	2.8 ± 1.4 [2.0, 3.2]	3.3 ± 1.2 [2.6, 4.2]	2.8 ± 1.4 [2.0, 3.2]	0.12
Volume (mm^3^)	114 ± 239 [14.1, 92.6]	110 ± 190 [36.2, 98.7]	115 ± 242 [13.9, 92.5]	0.94
CT attenuation (HU)	898 ± 347 [652, 1182]	574 ± 158 [496, 694]	921 ± 345 [678, 1187]	< 0.001

Values represent mean ± standard deviation. Interquartile range is given in squared brackets

### Dual-energy CT stone analysis

A total of 225 of 227 kidney stones were correctly classified by the dual-energy CT analysis. There was one false positive and one false negative uric acid stone classification. Pooled sensitivity and specificity were 1.0 (95%CI: 0.97, 1.00) and 0.93 (95%CI: 0.68, 1.00), respectively. The accuracy to discriminate a uric acid from a non-uric acid stone was 0.99 (95%CI: 0.97, 1.00). Eighty-two of 84 stones with a diameter of  ≤ 3 mm were correctly classified.

**Fig. 2:**
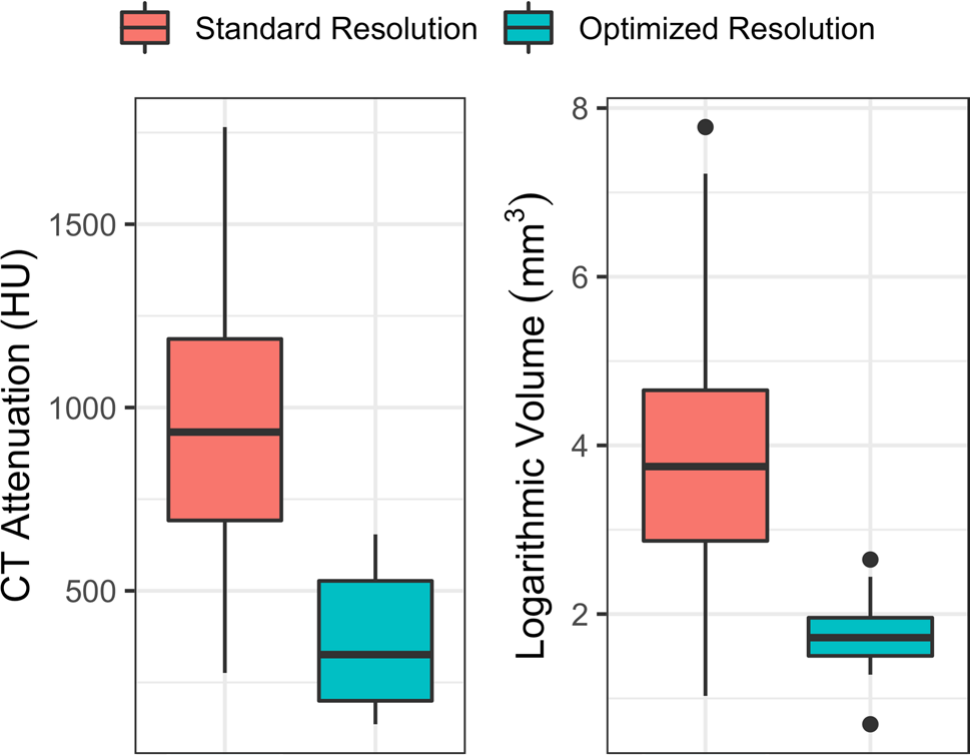
Stone characteristics for different DECT resolution. Note the increased CT attenuation and stone volume of stones imaged at standard resolution settings (resolution = 3) as compared to optimized resolution settings (resolution = 1)

**Fig. 3:**
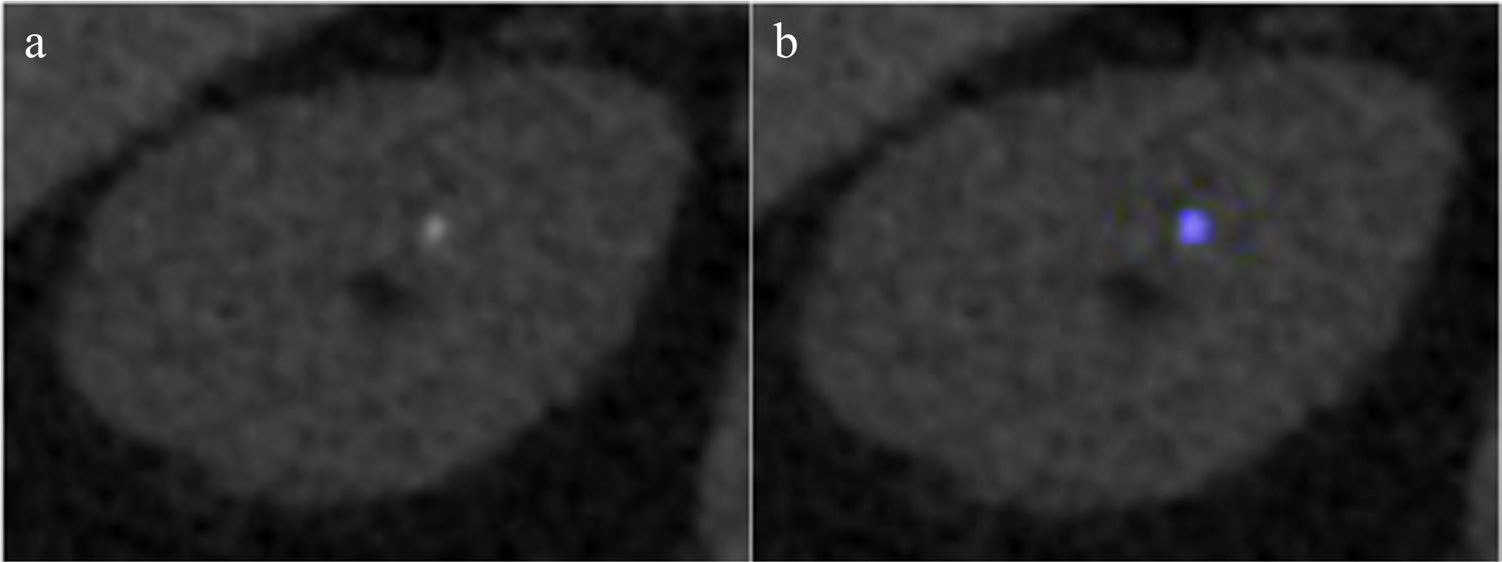
Effect of optimized resolution in small stones. **a** Initial dual-energy analysis failed at standard resolution in a stone of 2-mm diameter. **b** After optimizing the resolution parameter, the stone composition was correctly classified as a calcium oxalate

A total of 14 stones were not automatically color-coded by the DECT software algorithm. These stones were significantly smaller (mean length and width of 1.9 ± 0.3 mm and 1.6 ± 0.3 mm, respectively) and had a significantly lower CT attenuation (mean 372 ± 185 HU) as compared to the automatically detected stones (mean 932 ± 326 HU) (both *p* < 0.001) (Fig. 2). Thirteen of these stones were non-uric acid stones. After optimizing the resolution parameter, all 14 stones were color-coded (Fig. 3). Of these, 12 stones were correctly classified. The two falsely classified stones were small with a length and width of 2.2 mm × 1.6 mm and 2.3 mm × 1.9 mm, respectively. Both stones were imaged on the second-generation dual-source DECT (A).

## Discussion

This retrospective in vivo study investigated the diagnostic accuracy of low-dose dual-energy CT to discriminate uric acid from non-uric acid kidney stones with stone composition analysis as the reference standard. Imaging was performed on two different generations of dual-source dual-energy CT. Our results indicate a high diagnostic accuracy of low-dose DECT with a pooled sensitivity of 100%, specificity of 93%, and overall accuracy of 99%. This is in line with a meta-analysis regarding standard dose DECT which reported a mean pooled sensitivity of 95.5% and a specificity of 98.5% [10]. Several of the studies included in this meta-analysis were restricted to the limited availability of an ex vivo reference standard. Due to the availability of 227 kidney stones that were sent to stone composition analysis, our study adds to the available literature regarding the characterization of kidney stones using low-dose DECT.

Interestingly, 14 stones were not automatically encoded or classified using the standard resolution parameters of the DECT algorithm. These stones had significantly lower CT attenuation and were significantly smaller with an average length and width of 1.9 mm and 1.6 mm, respectively. This finding is comparable to former studies which reported a decreased detection of stones with a diameter of  < 3 mm [12, 14]. However, by optimizing the resolution parameter to a value of 1, the DECT algorithm was able to correctly classify 12 of these 14 stones. According to the vendor, the CT images are filtered prior to the analysis by the dual-energy algorithm. Here, the filter is the mean of a sphere with a radius of *r*. This radius *r* is equal to the resolution parameter, measured in voxels. Smaller values lead to a higher spatial resolution and improve the detection of small stones while larger values lead to smoothening of the images which can be advantageous in larger low-contrast stones. Overall, only two stones (one uric acid and one calcium oxalate – monohydrate stone) were falsely classified. These two stones were both small with a diameter of 2 mm and were both imaged on the second-generation dual-source DECT.

The results of our study indicate that low-dose DECT can be used with high accuracy in patients imaged for urolithiasis. To date, only a few studies have investigated the role of low-dose DECT in urolithiasis. Ascenti et al have investigated a combination of a low-dose single-energy CT scan and a stone-targeted DECT scan with a mean effective dose of 2.66 mSv [18]. However, such a combined protocol is difficult to implement if multiple stones are present and it is time-consuming, as a radiologist needs to be available to identify stones and determine the scan range of the DECT scan. Thomas et al reported high accuracy of low-dose DECT using a first-generation dual-source DECT with a mean effective dose of 2.7 mSv [21]. A very low radiation dose was achieved by Mahalingam et al in an Asian population using a second-generation dual-source DECT with a mean effective dose of 1.85 mSv [22]. Low-dose DECT imaging was achieved in our study with a mean effective dose of 2.43 mSv, being in the middle of these two aforementioned studies.

Due to the increasing demand for CT overall and in the emergency department, the optimization of radiation dose has become incrementally important in the last decades. While DECT suffered from an increased radiation dose as compared to single-energy CT in the first years after its introduction, multiple recent studies have demonstrated that DECT can be performed without a radiation dose penalty [23] or even at a lower radiation dose [24–26]. Therefore, it is particularly important to stress that our results indicate that DECT may be safely used as a surrogate for single-energy CT at low radiation dose with the additional benefit of stone characterization potentially impacting treatment options. A prospective randomized controlled trial is warranted to prove the non-inferiority of low-dose DECT.

The following limitations of our study merit consideration. First, this is a single-center study with a limited availability of different dual-energy CT approaches. Two different generations of dual-source DECT were investigated which are, however, widely used in clinical practice. Second, there was only a limited number of uric acid stones (7%). However, this reflects the prevalence of uric acid stones in industrialized countries (7–11%) [27, 28]. Third, there is a small bias of potential mismatch between the stones imaged and extracted in cases with multiple stones. To reduce this bias, spontaneously passed stones were omitted from the analysis, and stone characteristics (side, diameter, volume) were compared between the CT and urological extraction report. Furthermore, there was no patient in our study cohort in whom there were both uric acid and non-uric acid stones simultaneously present on the same side.

In conclusion, low-dose DECT demonstrated high accuracy to discriminate uric acid from non-uric acid stones even at small stone sizes. Future studies could investigate if low-dose DECT can replace low-dose SECT in a prospective randomized controlled non-inferiority trial.

## Abbreviations

CTDI_vol_: Volume CT dose index
DECT: Dual-energy CT
DLP: Dose length product
U-CT: CT to rule out urolithiasis

## Declarations

### Guarantor

The scientific guarantor of this publication is Olivio Donati.

### Statistics and biometry

One of the authors has significant statistical expertise.

### Methodology

• retrospective

• diagnostic study

• performed at one institution
